# Analytical methods for the determination of paracetamol, pseudoephedrine and brompheniramine in Comtrex tablets

**DOI:** 10.1186/s13065-019-0595-6

**Published:** 2019-07-02

**Authors:** Souha Hosam Youssef, Dalia Mohamed, Maha Abdel Monem Hegazy, Amr Badawey

**Affiliations:** 1 Pharmaceutical Analytical Chemistry Department, Faculty of Pharmacy, October University for Modern Sciences and Arts, 6th of October City, 11787 Egypt; 2 Analytical Chemistry Department, Faculty of Pharmacy, Helwan University, EinHelwan, Cairo, 11795 Egypt; 3 Analytical Chemistry Department, Faculty of Pharmacy, Cairo University, Kasr El-Aini Street, Cairo, 11562 Egypt; 4 Pharmaceutical Chemistry Department, Faculty of Pharmaceutical Sciences and Pharmaceutical Industries, Future University in Egypt (FUE), 90th street, fifth settlement, New Cairo, 11835 Cairo Egypt

**Keywords:** TLC, HPLC–UV, UPLC-MS/MS, Paracetamol, Brompheniramine maleate, Pseudoephedrine HCl

## Abstract

Comtrex^®^ tablets composed of paracetamol, pseudoephedrine and brompheniramine are widely used for relieving symptoms related to common cold. This study has overcome the challenging dosage form ratio (250:15:1) and proposed chromatographic methods for analyzing the ternary combination were utilized displaying different apparatus, solvents and sensitivity ranges. Three chromatographic methods namely thin layer chromatography (TLC), high performance liquid chromatography with ultra-violet detection (HPLC–UV) and ultra-performance liquid chromatography coupled to tandem mass spectrometry (UPLC-MS/MS) were developed and validated for the simultaneous determination of the three drugs. Concerning the TLC method, aluminum TLC plates pre-coated with silica gel 60F_254_ were used and methanol:water:ammonia (9:1:0.1, v/v/v) was applied as a mobile phase; scanning of the plates was carried out at 254 nm. For the HPLC–UV method C_18_ column was used with an isocratic elution mobile phase composed of water:acetonitrile (75:25, v/v; pH 3.2) and the detection was at 210 nm. For the UPLC-MS/MS method; separation was performed on a UPLC-BEH C_18_ column with methanol: 0.1% ammonium formate (60:40, v/v) as the mobile phase utilizing diphenhydramine as an internal standard and mass spectrometry was used for detection. The methods were simple, sensitive, accurate and precise. Statistical analysis revealed no significant difference from the reported methods in regard to accuracy and precision.

**Electronic supplementary material:**

The online version of this article (10.1186/s13065-019-0595-6) contains supplementary material, which is available to authorized users.

## Introduction

The demand on effective and efficient over the counter medication is increasing everyday leading pharmaceutical companies to include more components in their dosage forms. Therefore, economic, fast and accurate methods for analyzing such mixtures are needed. Comtrex^®^ Maximum Strength tablets is a ternary combination of paracetamol (PAR), pseudoephedrine hydrochloride (PSE) and brompheniramine maleate (BRM). It is available in the Egyptian market and is widely used for relieving symptoms of the flu: light pain, headache, sore throat pain, pyrexia, congested nose and sneezing.

**Fig. 1:**
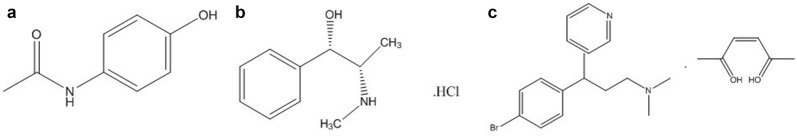
Chemical structures of: **a** paracetamol, **b** pseudoephedrine HCl and **c** brompheniramine maleate

PAR; *N*-(4-hydroxyphenyl) acetamide reduces pain and fever used in the treatment of arthritis, tooth ache and headaches [1]. It is a major constituent in many cold and flu medications. PAR can also be administered for controlling intolerable pain (namely; post-operative pain) specifically when combined with non-steroidal anti-inflammatory drugs or opioid analgesics [2]. Paracetamol is official in the British pharmacopoeia (BP) [3]. Literature survey has revealed that PAR in its single form or combined with other drugs was determined by titrimetry [3, 4], spectrophotometry [5–7], spectrofluorimetry [8], high performance thin layer chromatography (HPTLC) [9, 10] GC–MS [11], HPLC–UV [11–13], HPLC–MS/MS [14] and micellar electrokinetic capillary chromatography (MECC) [15]. PSE; [(+)-threo-a-[1-methylamino) ethyl] benzyl alcohol] hydrochloride, is a sympathomimetic amine that acts on adrenergic receptors directly. It is often used for bronchodilation and treating nasal congestion by shrinking the swollen nasal mucous membranes [16]. PSE is official in the BP [3]. Several methods were found in the literature for its quantitation such as titrimetry [3], spectrophotometry [17, 18], HPTLC [19, 20],GC [21], Micellar electrokinetic chromatography (MEKC) [22], HPLC–UV [23, 24] and capillary electrophoresis (CE) [25]. BRM; (3RS)-3-(4-Bromophenyl)-*N*,*N*-dimethyl-3-(pyridin-2-yl)propan-1-amine(Z)-butenedioate, is an antihistamine used for relieving allergy symptoms such as sneezing, itching and watery eyes [26]. BRM is official in the BP [3]. Being a recently released drug; only two methods were found in the literature for BRM determination combined with phenylephrine and in blood plasma [26, 27]. In addition, the BP has described a titrimetric method for determining its concentration [3]. The structures of the three drugs are demonstrated in Fig. 1.

The determination of cough and cold pharmaceutical preparations is usually challenging as these preparations are composed of complex formulae comprising numerous active constituents and a wide range of additives. The separation of these ingredients in the pharmaceutical dosage forms is difficult due to similarities of their physical and chemical properties. Thus, our aim was to conduct different sensitive, accurate and precise chromatographic methods (TLC, HPLC–UV and UPLC-MS/MS) for the separation and quantitation of PAR, PSE and BRM in their combined dosage form specifically as to the extent of our knowledge; from a detailed literature survey that only one chemometric method [28] was reported for their simultaneous determination. In addition, was to demonstrate the privileges introduced by each method and to conduct statistical comparison between the newly developed methods and reported ones to assure the applicability of our methods for their intended use.

## Experimental

### Apparatus and software

#### TLC-densitometric system

The TLC measurements were carried out using: a CAMAG TLC Scanner 3 S/N 130,319 operated with win CATS software, Linomat 5 autosampler (CAMAG, Muttenz, Switzerland), CAMAG micro syringe (100 μL) and TLC aluminum sheets (20 × 20 cm) pre-coated with silica gel 60 F_254_ (Merck, Darmstadt, Germany) were utilized.

#### HPLC–UV system

The HPLC experiments were performed on: Agilent 1200 series chromatographic system encompassed with a quaternary pump, a micro vacuum degasser, a thermostatted column compartment and a variable wavelength UV–VIS detector. In addition, Agilent 1200 series autosampler was used for sample injection. Agilent ChemStation software, version A.10.01 was utilized to collect and process data. Separation was performed on Agilent Zorbax SB-C_18_ (150 × 4.6 mm, 5 μm) column which is manufactured by Agilent Technologies (Polo Alto, CA, USA). To adjust the pH, a “Jenway 3505” pH-meter (Jenway, UK) was used.

#### UPLC-MS/MS system

A XEVO-TQD triple stage quadrupole mass spectrometer, Waters (Singapore), equipped with an electrospray ionization (ESI) source was utilized to analyze the mixture. The Waters Acquity UPLC system has included an Acquity quaternary solvent manager (QSM pump) and an Acquity sample manager-FTN, Waters (Singapore), operated at room temperature. An Acquity UPLC-BEH C_18_ column (50 × 2.1 mm, 1.7 μm) from Waters (Singapore) was used. Data gathering and processing were implemented using MassLynx software (Support ID MS1HAO1637).

### Chemicals and reagents

#### Pure samples

GlaxoSmithKline (Cairo, Egypt) has generously provided PAR, PSE and BRM. According to reported methods of analysis their purities were found to be 99.40 ± 0.778 [29], 100.11 ± 0.427 [29] and 99.12 ± 0.699 [26] for PAR, PSE and BRM, respectively. Diphenhydramine (IS) was kindly given by Sigma Pharmaceutical Industries, Steinheim, Germany.

#### Market sample

Comtrex^®^ Maximum Strength coated tablets label claims to contain 500 mg of PAR, 30 mg PSE and 2 mg BRM (Batch number: A514875), manufactured by GlaxoSmithK1ine Egypt for Novartis Pharma Egypt, under license from Novartis Consumer Health, Switzerland and it was bought from the local market.

#### Solvents

Methanol (HPLC grade) used for TLC was purchased from Fischer Scientific UK Ltd (Loughborough, UK), while methanol (Ultra-gradient HPLC grade) used for UPLC-MS/MS was purchased from J.T.Baker (Amsterdam, The Netherlands). Acetonitrile was obtained from Tedia (Fairfield, USA). Ammonia and formic acid were obtained from Scharlau chemicals (Barcelona, Spain). Phosphoric acid was acquired from El-Nasr Pharmaceutical Chemicals Co. (Cairo, Egypt). Double distilled deionized water was purchased from Otsuka (Cairo, Egypt).

### Standard solutions

#### For TLC method

Separate stock standard solutions of (5.0 mg/mL) PAR, (5.0 mg/mL) PSE and (4.0 mg/mL) BRM were prepared in water. The same solvent was used for further dilution in order to prepare the working standard solutions with the concentrations of (2.5 mg/mL) PAR, (2.5 mg/mL) PSE and (2.0 mg/mL) BRM.

#### For HPLC method

Separate stock standard solutions of (1.0 mg/mL) of each of PAR, PSE and BRM were prepared using water as a solvent. Then the same solvent was used for further dilution in order to prepare (0.5 mg/mL) working standard solutions of the three drugs.

#### For UPLC-MS/MS method

Separate stock standard solutions (100.0 µg/mL) of PAR, PSE and BRM were prepared in methanol. Then the same solvent was used for further dilution in order to prepare (5.0 µg/mL) working standard solutions of the three drugs.

### Chromatographic and mass spectrometric conditions

#### Chromatographic conditions for TLC-densitometric method

The samples were added to TLC sheets in the form of bands represented as 10 µL/band using a 100-µL syringe (The width of each band was 6 mm; the bands were about 1 cm apart from each other and at least 1 cm away from the bottom edge of the plate). The developing system was methanol:water:ammonia (9:1:0.1, v/v/v). The developing system was left in the chromatographic tank for about 1 h at room temperature for saturation with the solvents used. Then linear ascending separation was performed to a distance of about 8 cm from the lower edge of the TLC plate. Consequently, the plates were air dried and scanned at 254 nm. The detection was carried out with the aid of CAMAG TLC Scanner 3 functioned in the absorbance mode using a deuterium lamp as the source of radiation while keeping the slit dimension at 3 mm × 0.45 mm and the speed of scanning at 20 mm/s.

#### Chromatographic conditions for HPLC–UV method

RP-HPLC was performed at room temperature using a ZorbaxSB-C_18_ column (150 × 4.6 mm, 5 μm). The utilized mobile phase consisted of water:acetonitrile (75:25, v/v, pH 3.2) where the pH adjustment was achieved using phosphoric acid. Filtration of the mobile phase was carried out before injection using 0.45 μm Millipore membrane filter (Billerica, MA). The flow rate was 0.7 mL/min with 20 μL injection volume and the signals were detected at 210 nm. The total run time was about 4 min.

#### Chromatographic conditions for UPLC-MS/MS method

Chromatographic separation was accomplished on UPLC-BEH C_18_ column (50 × 2.1 mm, 1.7 µm). The mobile phase contained methanol: 0.1% ammonium formate (60:40, v/v) which was filtered before use on 0.45 μm Millipore membrane filter (Billerica, MA). The flow rate was 0.3 mL/min with 2 µL injection volume. The duration of the analysis was about 2 min. Diphenhydramine was used as an internal standard (IS).

#### Mass spectrometric conditions for UPLC-MS/MS method

The mass spectrometric detection was accomplished utilizing electrospray ionization (ESI) which was operated in the positive-ion. The optimized parameters are: cone gas flow of 15 L/h, desolvation gas flow of 500 L/h, source temperature of 150 °C, desolvation temperature of 400 °C, cone voltage of 20 V and capillary voltage 3 kV. The quadrupole mass spectrometer was set at the MRM mode, to monitor the following transitions (molecular ions/product ions): PAR m/z 152.03/110.26, PSE 166.11/148.13, BRM 318.971/274.04 and diphenhydramine (IS) 255.75/166.15 using collision energy of 24, 10, 15 and 10 eV, respectively.

### Procedures

#### Construction of calibration curves

##### For TLC method

Different aliquots of PAR (1.0–9.6 mL), PSE (0.4–9.0 mL) and BRM (0.1–7.5 mL) were separately and accurately transferred from their corresponding working standard solutions into 10-mL volumetric flasks and the volume was completed with water to produce series of concentrations of 250.0–2400.0 µg/mL for PAR, 100.0–2250.0 µg/mL for PSE and 20.0–1500 µg/mL for BRM. From each concentration 10 µL were spotted on to the TLC plates which were developed using methanol:water:ammonia (9:1:0.1, v/v/v). After applying all the chromatographic conditions previously described; the densitograms were recorded at 254 nm. Calibration curves represented by the peak area ratio (10, 15 and 1.2 µg/band of PAR, PSE and BRM, respectively) were used as an external standard) against the corresponding concentration of the three drugs were plotted and the regression equations were calculated. The linearity ranges are 2.5–24.0 μg/band for PAR, 1.0–22.5 μg/band for PSE and 0.2–15.0 μg/band for BRM.

##### For HPLC method

Different aliquots of PAR (0.1–2.0 mL), PSE (0.6–4.0 mL) and BRM (0.6–4.0 mL) were separately and accurately transferred from their respective working standard solutions into 10-mL volumetric flasks and completed to volume with the mobile phase to prepare series of concentrations of 5.0–100.0 µg/mL for PAR, 30.0–200.0 µg/mL for PSE and 30.0–200.0 µg/mL for BRM. All the chromatographic conditions were applied then scanning was performed at 210 nm. The calibration curves were then created where the peak area ratio (the found peak area to that of a standard of the same drug) of each drug was plotted versus the corresponding concentrations using 5.0, 30.0 and 30.0 µg/mL of PAR, PSE and BRM, respectively as external standards. Finally, the regression equations were computed. The calibration curves were constructed in the following ranges 5.0–100.0 µg/mL for PAR, 30.0–200.0 µg/mL for PSE and 30.0–200.0 µg/mL for BRM.

##### For UPLC/MS–MS method

Separate aliquots were accurately transferred from PAR (80.0–2000.0 µL), PSE (12.0–1000 µL) and BRM (8.0–1000.0 µL) working standard solutions into three series of 10-mL volumetric flasks followed by the addition of 10 ng/mL of the IS on each concentration. Finally, methanol was utilized to complete the volume, thus, solutions of different concentrations were prepared. Then 2 µL aliquots of each solution were injected onto the UPLC-MS/MS system after being filtered through membrane filter (0.45 mm). The previously described chromatographic conditions were applied. Subsequently, the calibration curves were plotted for each drug by utilizing the peak area ratios of each drug to that of the IS versus the equivalent concentrations. Then the regression equations were computed. The linearity ranges were as follows 40.0–1000.0 ng/mL for PAR, 6.0–500.0 ng/mL for PSE and 4.0–500.0 ng/mL for BRM.

#### Assay of laboratory-prepared mixtures

Different aliquots of the PAR, PSE and BRM were accurately transferred from their working standard solutions and used for the preparation of various laboratory prepared mixtures of different ratios. The chromatographic conditions for each proposed method were applied on the prepared mixtures. The concentrations of the drug were computed from their corresponding regression equation where the mean of three experiments was used.

#### Application to pharmaceutical formulation

Ten Comtrex^®^ maximum strength tablets were weighted accurately, ground and blended well. An amount equal to one tablet was weighed and taken into a beaker; the three components were extracted with (3 × 30 mL) water for TLC and HPLC–UV methods and with methanol for UPLC/MS–MS method. Then sonication was carried out for 15 min (for each extraction). The solution was filtered into a 100-mL volumetric flask and the same solvents were utilized to complete the volumes, thus (Stock 1) was obtained with the following concentrations 5000.0 µg/mL of PAR, 300.0 µg/mL of PSE and 20.0 µg/mL of BRM.

*For the TLC method* Stock 1 was directly used for PSE and BRM determination. For PAR determination; an aliquot of 20 mL was accurately transferred from Stock 1 into a 100-mL volumetric flask and completed to volume with water where a solution of 1000 µg/mL of PAR was obtained.

*For the HPLC–UV method* An aliquot of 0.5 mL was accurately transferred from Stock 1 into a 100-mL volumetric flask followed by spiking with 5000 µg of each of PSE and BRM and the mobile phase was utilized to complete the volume. Thus, the following concentrations were attained 25.0 µg/mL of PAR, 51.5 µg/mL of PSE and 50.1 µg/mL of BRM.

*For UPLC method* An aliquot of 1 mL was accurately transferred from Stock 1 into a 100-mL volumetric flask and completed to volume with methanol to obtain Stock 2. Further, a volume of 2 mL from Stock 2 was accurately taken into a volumetric flask (100-mL) and completed to volume with methanol to obtain a solution with the following concentration of 1000 ng/mL of PAR, 60 ng/mL of PSE and 4 ng/mL of BRM.

The procedures described under “Construction of calibration curves” were carried out for each of the suggested methods. The concentration of each drug was calculated by substituting in its corresponding regression equations. For the HPLC–UV method the concentration of PSE and BRM which is claimed to be found was calculated after subtraction of the spiked amount. The standard addition technique was also performed by the addition of different known amounts of the pure standard drugs to the pharmaceutical dosage form before continuing the previously mentioned procedures.

## Results and discussion

Resolving ternary mixtures is an ordeal due to the interference of the components in the same dosage form. As revealed by the literature review, no chromatographic attempts were reported to analyze this mixture. Chromatographic methods are usually of choice due to higher sensitivity, accuracy and efficiency. The three proposed methods have offered flexibility regarding the use of different chromatographic apparatus, detectors and principles. The methods have also shown different linearity ranges and different mobile phases providing different options for analysis. Validation was achieved based on the ICH guidelines [30].

### Method development

#### TLC

**Fig. 2:**
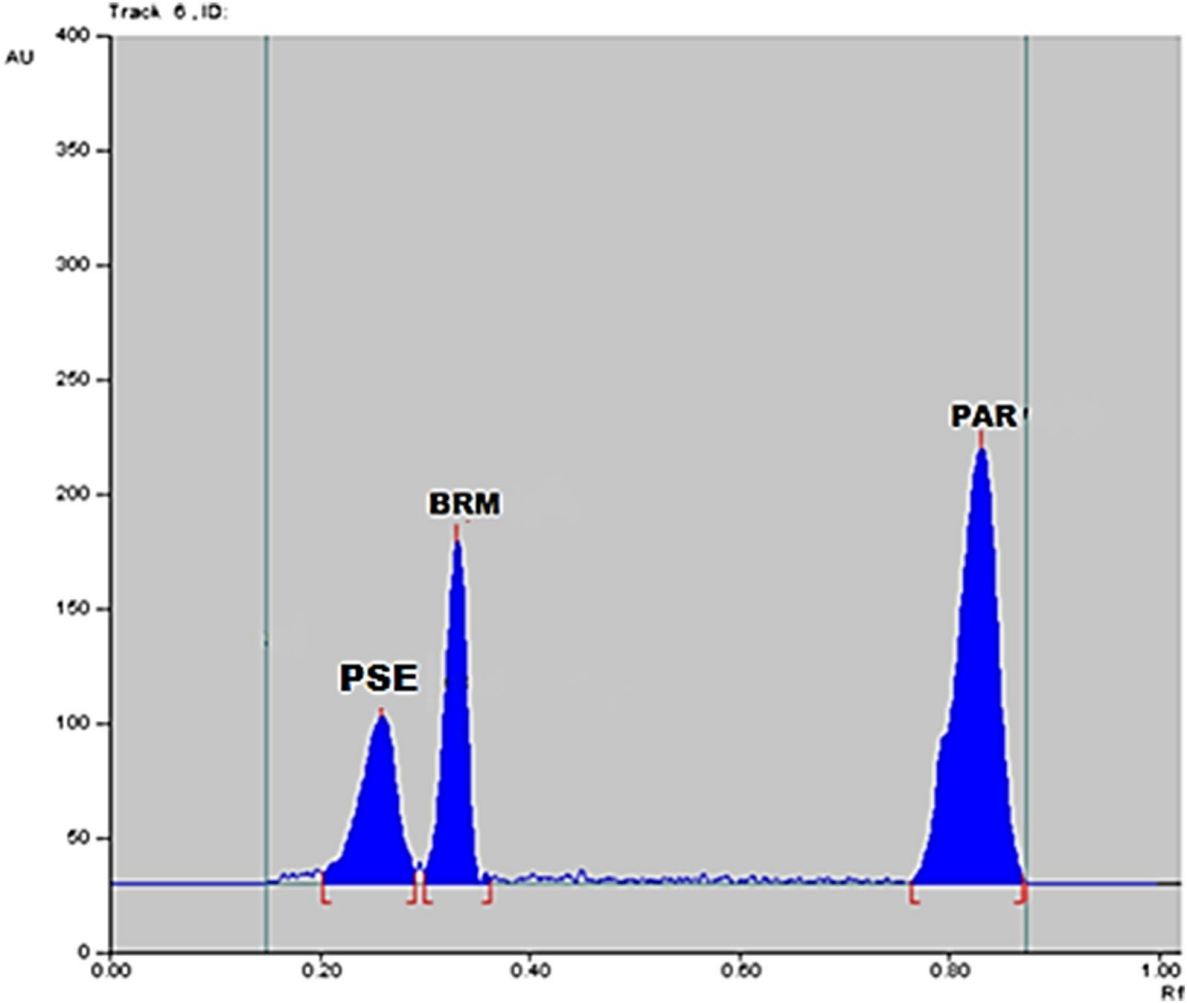
TLC chromatogram of separated peaks of pseudoephedrine, brompheniramine maleate and paracetamol, R_f_ = 0.24, 0.32 and 0.81, respectively; using methanol:water:ammonia (9:1:0.1, v/v/v) as a mobile phase

This method has presented a simple approach to separate and quantify PAR, PSE and BRM directly on TLC plates by determining the optical density of the separated bands. In order to optimize the proposed method, different developing systems were tried to obtain good separation of the three drugs. First, chloroform:acetone:ammonia (8:2:0.1, v/v/v) was applied but none of the three components were separated. A second trial using different solvents was carried out using methanol:chloroform (8:2, v/v), PAR was separated but PSE and BRM were not resolved despite trying different ratios of the two solvents. Chloroform was replaced with water and different ratios of methanol:water were tried, the trials included (8:2, v/v) where PAR and BRM were separated but PSE was tailed. Other trials including methanol:ammonia were applied. The ratio of methanol:ammonia (10:1, v/v), was successful in separating PSE and BRM but PAR appears with the solvent front. Finally, methanol:water:ammonia (9:1:0.1, v/v/v) has offered the best resolution with sharp symmetrical peaks. Band width of 6 mm was selected in order to reduce band diffusion. Moreover, the spectra of the 3 drugs were measured separately and accordingly scanning was attempted using 210, 230, 254 and 265 nm, where the 3 drugs showed high absorbance values the scanning wavelength was selected to be 254 nm as it has resulted in sharp and symmetrical peaks with minimal level of noise. The R_f_ values were found to be 0.24, 0.32 and 0.81 for PSE, BRM and PAR, respectively. A typical chromatogram is displayed in Fig. 2, from which it was obvious that each of the three drugs could be determined without any interfering signals from the other. The TLC method has demonstrated the advantages of utilizing simple developing systems which do not require pH adjustments. Additionally, many samples can be run simultaneously with the consumption of low volumes of the mobile phase, thus offering minimum analysis time and consequently cost effective.

#### HPLC–UV

This method has allowed for the separation and quantification of PAR, PSE and BRM. In order to get the best separation of the drugs, it was necessary to adjust different parameters as the mobile phase, column, scanning wavelength, and flow rate.

Several attempts using different solvent mixtures were applied before reaching optimal separation. First, combinations of methanol:water were tried (50:50, 25:75 and 15:85, v/v). However, the trials were not successful and the 3 drugs were eluted in overlapping retention times. Afterwards, acetonitrile was used instead of methanol and acetonitrile:water in the ratio 50:50, v/v was applied as a mobile phase. Resolution was improved where PAR and PSE provided 2 peaks. The ratio of the promising combination was gradually altered to elute BRM without overlapping with PAR and PSE. Finally, the three drugs were separated by the ratio 25:75, v/v, acetonitrile:water, respectively. The mobile phase was adjusted at three different pH values; 3, 5 and 8 to select the optimum pH for the developed method. It was observed that in basic media, the peaks suffered from broadening and tailing. In addition, trifluoroacetic acid, acetic acid and phosphoric acid were tried for pH adjustment and no difference was evident, regarding peak sharpness and retention times. Consequently, phosphoric acid was selected due to its availability, low price and its ability to adjust the desired pH using small amounts.

**Fig. 3:**
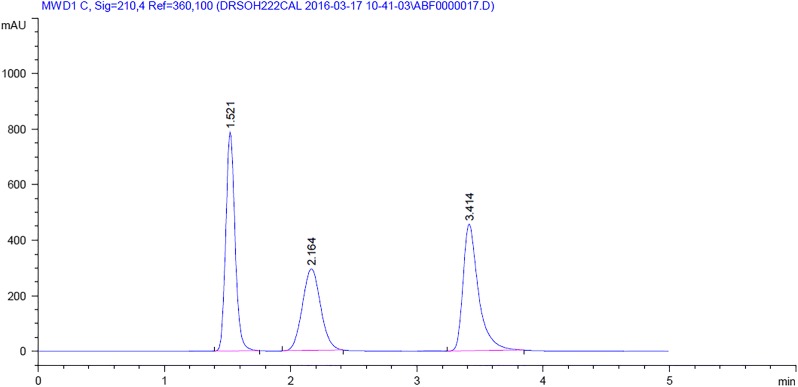
HPLC chromatogram showing the separation of a mixture composed of pseudoephedrine HCl (t_R_ = 1.521), brompheniramine maleate (t_R_ = 2.164) and paracetamol (t_R_ = 3.414) using the mobile phase, water:acetonitrile (75:25, v/v, pH 3.2)

The best peak shape and resolution as well as a reasonable linearity range were achieved upon using a ZorbaxSB-C_18_ column (150 × 4.6 mm, 5 μm) with an isocratic elution mobile phase composed of water:acetonitrile (75:25, v/v) where phosphoric acid was utilized to adjust pH at 3.2 and keeping the flow rate at 0.7 mL/min. Detection was carried out at several wavelengths; 210, 230, 254 and 265 nm but the sharpest peaks and optimum peak area was achieved at 210 nm. These conditions have permitted the complete separation of the investigated drugs within 4 min with the retention times of 1.521 min, 2.164 min and 3.414 min for PSE, BRM and PAR respectively, as demonstrated in Fig. 3.

The acid dissociation constant (Ka) of the analytes plays an important role in determining their elution order. The lower the pKa value, the higher the tendency of the drug to be ionized and as a result more polar and rapidly eluted from the reverse phase column. By observing the pKa values of the 3 drugs; 9.50, 9.48 and 9.22 for PAR, PSE and BRM, respectively, the elution order could be predictable. PSE with the lowest pKa value was eluted first, followed by BRM then PAR with the highest pKa. However, as the pKa of the 3 drugs are very close, their order of elution was ascertained by the optimization of the chromatographic conditions. Moreover, lipophilicity (represented by the partition coefficient, LogP) of the three drugs were studied since it is the second factor guiding chromatographic separation. The values of Log P for PAR, PSE and BRM were found to be 0.51, 1.32 and 3.75, indicating that PAR is least lipophilic drug and BRM is the most. Consequently, the expected elution order is PAR, followed by PSE and BRM eluted last. That being said, practically this order was not obtained. Nonetheless, this order was confirmed by peaks purity and injecting each standard drug solution separately.

The proposed HPLC method has offered several advantages as the absence of buffer in the mobile phase which will save the efficiency of the column thus increasing its lifetime. The very short run time and the low flow rate have reduced the volume of the organic solvents used in the mobile phase which is considered of high economic value when the method is utilized for routine work. Additionally, the method has demonstrated good sensitivity where it was capable of quantifying the drugs in the concentration ranges of 5.0–100.0 µg/mL for PAR, 30.0–200.0 µg/mL for PSE and 30.0–200.0 µg/mL for BRM.

#### UPLC-MS/MS

**Fig. 4:**
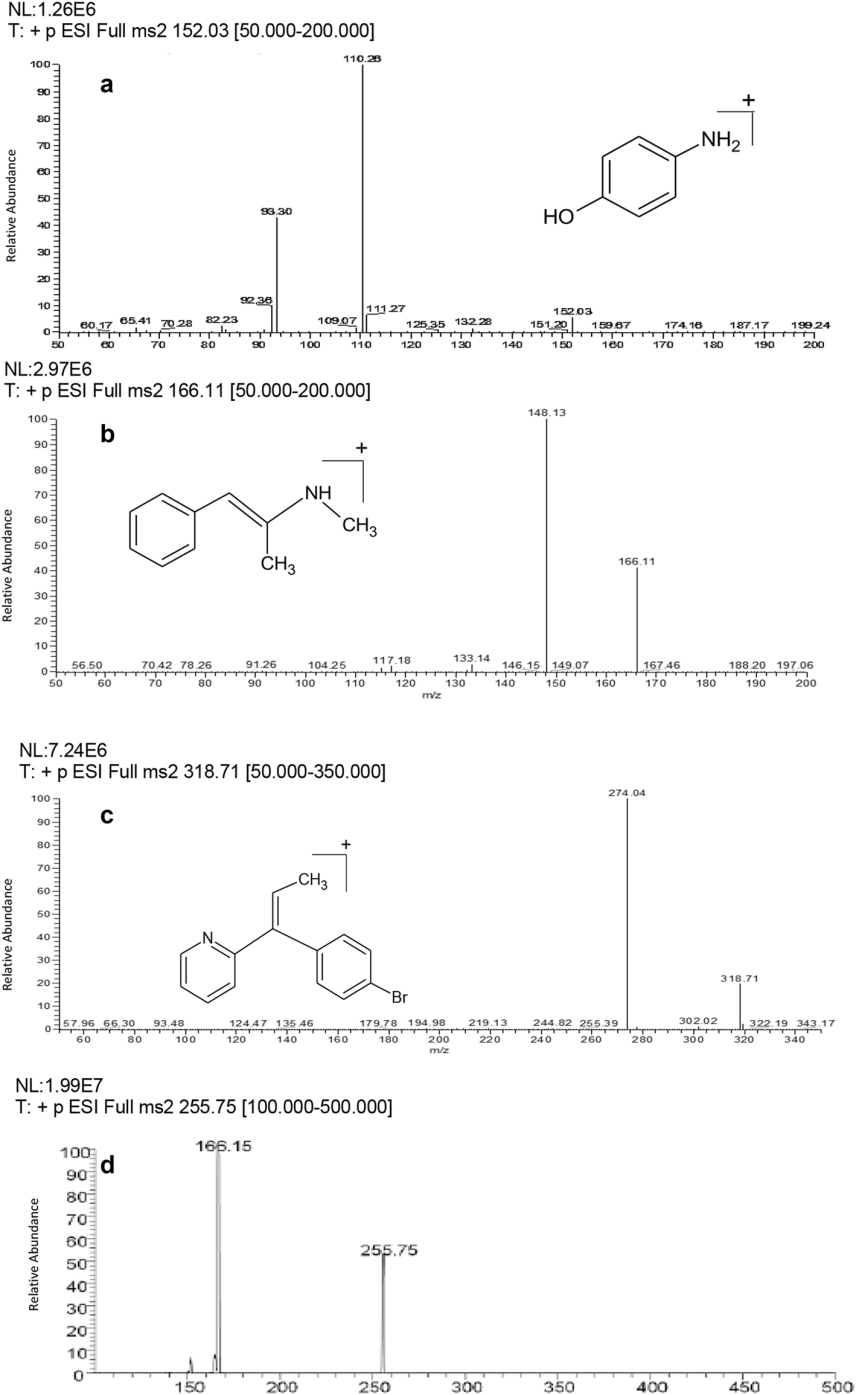
Product scan mass spectra of **a** paracetamol, **b** pseudoephedrine hcl, **c** brompheniramine maleate, **d** diphenhydramine

The UPLC-MS/MS method has demonstrated a successful trial for the quantification of PAR, PSE and BRM in the nano-gram level. Both chromatographic and mass spectrometric conditions were optimized. Regarding the mass spectrometry; 1.00 µg/mL neat solutions of the drugs and IS were infused into the mass spectrometer in the range of 100–400 *m/z* so as to adjust the detection of both the precursor ions and product ions utilizing positive electrospray ionization technique. The positive polarity mode was preferred due to the ability of the studied drugs to be protons acceptors leading to the highest abundance of both the precursor and the product ions. The protonated molecular ions [M + H]^+^ of PAR, PSE, BRM and the internal standard were detected on the full scan mass spectra with the masses of 152.03, 166.11, 318.71 and 255.75 *m/z*, respectively. The application of adequate collision energy in Q2 has resulted in the production of significant fragments. The MS/MS transition 152.03 → 110.26, 166.11 → 148.13, 318.71 → 274.04 and 255.75 → 166.15 for PAR, PSE, BRM and the internal standard, respectively, were chosen as these products ions represented the most abundant ones (Fig. 4). Moreover, the capillary temperature and sheath gas flow were optimized due to their role in reduction of the ion suppression and alteration of the sensitivity.

**Fig. 5:**
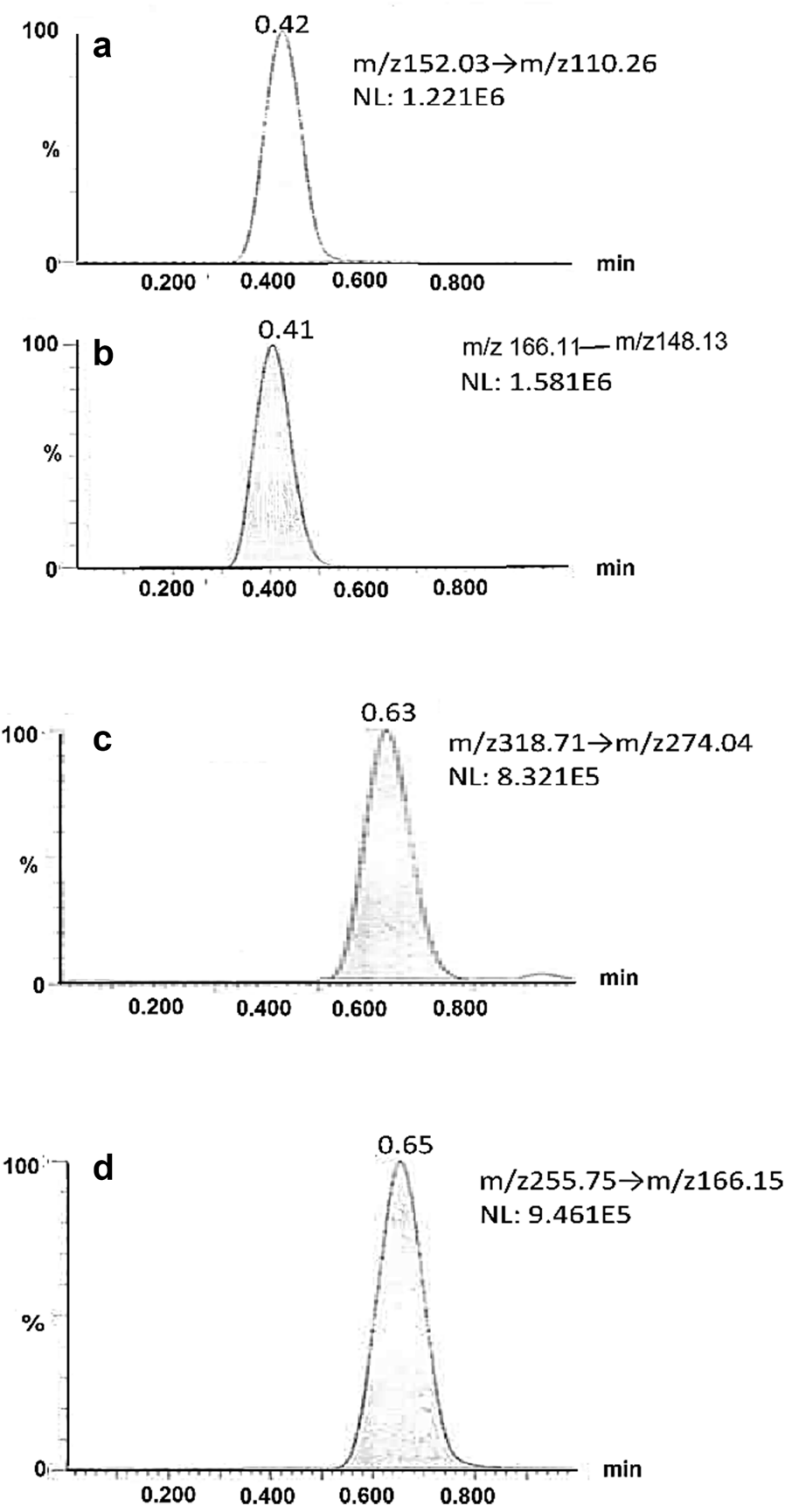
UPLC-MS/MS chromatogram for the separation of **a** paracetamol, **b** pseudoephedrine, **c** brompheniramine maleate and **d** diphenhydramine

For the chromatographic conditions, the analysis of the three drugs and the internal standard was performed using isocratic elution with the objective of developing a simple separation procedure. Various combinations of methanol and ammonium formate solution in varying ratios of each component were tried, however, the most appropriate mobile phase consisted of methanol: 0.1% ammonium formate (60:40, v/v) as it has allowed for both protonation and fast elution of the three drugs. Ammonium formate has aided in attaining good response for positive mode MS detection. On the other hand, the high proportion of methanol (60%) in the mobile phase has facilitated the elution of the drugs within 1 min with a relatively low flow rate of 300 μL/min. The UPLC-BEH C_18_ column (50.0 mm × 2.1 mm, 1.7 μm) has resulted in good peak shapes for the investigated drugs. The chromatogram of the drugs is displayed in Fig. 5. The proposed UPLC-MS/MS has demonstrated several advantages where the use of the small column size with small particle size has resulted in rapid analysis time (1 min) with well identified peaks. The flow rate of 300 µL/min has obviously resulted in low consumption of solvent which cuts the running costs of the analysis which is beneficial especially for quality control laboratories. Finally, the method has offered very high sensitivity as it was capable of quantifying the drugs in the nano-gram level within the following ranges 40.0–1000.0 ng/mL for PAR, 6.0–500.0 ng/mL for PSE and 4.0–500.0 ng/mL for BRM.

### Method validation

The developed procedures were validated based on the ICH Q2 (R1) recommendation [30].

#### Linearity

The linearity of PAR, PSE and BRM was estimated by investigating six concentrations from each drug in triplicates using the optimum chromatographic conditions for each method as well as the mass spectrometric parameters for the UPLC-MS/MS method. A linear relationship was observed between the peak area ratios of each drug (External standard was used for the TLC and HPLC methods, while internal standard was used for UPLC-MS/MS method) and their equivalent concentrations. The linearity ranges varied between the methods and they were found to be 2.5–24.0 μg/band, 1.0–22.5 μg/band and 0.2–15.0 µg/band for the TLC method, 5.0–100.0 µg/mL, 30.0–200.0 µg/mL and 30.0–200.0 µg/mL for the HPLC method and lastly 40.0-1000.0 ng/mL, 6.0–500.0 ng/mL and 4.0–500.0 ng/mL for the UPLC-MS/MS method for PAR, PSE and BRM, respectively. The linear relationship of the calibration curves were confirmed by the values of the correlation coefficients which were approaching unity. The calibration equations, standard deviation of the slope and standard error of the intercept are abridged in Table 1.

**Table 1:** Regression and validation parameters of the proposed chromatographic methods for determination of PAR, PSE and BRM

Parameters	TLC			HPLC–UV			UPLC-MS/MS		
	PAR	PSE	BRM	PAR	PSE	BRM	PAR	PSE	BRM
Linearity range	2.5–24.0 µg/band	1.0–22.5 µg/band	0.2–15.0 µg/band	5.0–100.0 µg/mL	30.0–200.0 µg/mL	30.0–200.0 µg/mL	40.0–1000.0 ng/mL	6.0–500.0 ng/mL	4.0–500.0 ng/mL
Slope	0.0386	0.0515	0.8877	0.1178	0.03150	0.0364	0.03896	0.1133	0.0030
Intercept	0.5351	0.3440	0.1344	0.4580	− 0.01384	− 0.0039	− 0.9660	2.0740	1.0370
S_y/x_	0.0062	0.0043	0.0074	0.0319	0.0546	0.0684	0.1182	0.0202	0.0004
Correlation coefficient (r)	0.9999	1.0000	1.0000	1.000	0.9998	0.9997	0.9997	1.0000	1.0000
Mean^a^	99.46	99.74	100.02	100.67	99.02	100.32	99.55	98.99	98.28
SD	1.57	1.09	0.74	1.43	1.17	1.52	1.27	1.11	0.91
LOD	0.53	0.28	0.03	0.893	5.72	6.21	10.01	0.59	0.44
LOQ	1.61	0.84	0.08	2.71	17.33	18.79	30.34	1.78	1.33
Precision (RSD %)^ab^	0.36	0.45	0.40	0.31	0.29	0.58	0.27	0.36	0.33
Repeatability Intermediate precision	1.08	0.99	0.93	0.83	1.15	1.19	1.03	1.53	1.48
Robustness	1.68	1.52	1.26	1.25	1.55	1.67	1.34	0.13	0.03

^a^Average of three experiments

^b^Relative standard deviations (RSD%) of three concentrations, the concentration were as follows: For TLC; PAR (6.0, 10.0, 18.0 µg/band), PSE (5.0, 10.0, 15.0 µg/band) and BRM (0.8, 5.0, 12.0 µg/band), for HPLC; PAR (10.0, 30.0, 50.0 µg/mL), PSE (50.0, 100.0, 150.0 µg/mL) and BRM (0.80, 100.0, 150.0 µg/mL) and for UPLC-MS/MS; PAR (70.0, 120.0, 200.0 ng/mL), PSE (20.0, 50.0, 200.0 ng/mL) and BRM (20.0, 40.0, 100.0 ng/mL)

#### Accuracy

The accuracy of the three suggested methods was evaluated through the analysis of different concentrations of PAR, PSE and BRM where each concentration was performed three times using the previously described procedures. The accuracy in terms of percentage recoveries (mean) as well as standard deviation is demonstrated in Table 1, where the obtained results has assured the appropriate accuracy of the developed methods.

#### Precision

The intraday precision was assessed by analyzing three concentrations of the drugs in triplicates during the same day. However, the interday precision was accomplished by analyzing the same three concentrations of the drugs in triplicates on three successive days. The precision expressed as percentage relative standard deviations (RSD %) was calculated as shown in Table 1. It was clear from the results that the RSD% has not exceeded 2% for all concentrations demonstrating that all the proposed methods could be considered as precise methods.

#### Specificity

The specificity of a method is measured by its ability to quantify a certain analyte despite the presence of other interfering substances in a mixture or matrix. Thus, the specificity of the three proposed methods was verified through the analysis of six laboratory prepared mixtures composed of different amounts of PAR, PSE and BRM in addition to the analysis of Comtrex^®^ Maximum Strength tablets using the previously described procedures.

The chromatograms of the different mixtures as well as the dosage form showed well resolved peaks for the three drugs, where the chromatograms obtained from the sample solutions of the drugs (laboratory mixtures and dosage form) were identical to those obtained from the standard solution of pure authentic powder. Moreover, no extra peaks were detected in the chromatograms obtained from the analysis of the dosage form indicating that the excipients present in the tablets have not interfered in the analysis procedure. The data abridged in Tables 2 and 3 were satisfactory showing good percentage recoveries and standard deviation proving the specificity of the proposed methods.

**Table 2:** Analysis of laboratory prepared mixtures by the proposed chromatographic methods

Mix	TLC						HPLC–UV						UPLC-MS/MS					
	Concentration			Recovery %^a^			Concentration			Recovery %^a^			Concentration			Recovery %^a^		
	PAR	PSE	BRM	PAR	PSE	BRM	PAR	PSE	BRM	PAR	PSE	BRM	PAR	PSE	BRM	PAR	PSE	BRM
1	6.0	5.0	0.8	98.54	99.80	102.53	100.0	30.0	100.0	101.09	101.46	100.29	40.0	20.0	20.0	98.44	99.13	99.96
2	2.5	10.0	0.4	98.00	99.70	99.25	50.0	100.0	50.0	99.66	101.30	101.64	400.0	50.0	50.0	100.81	98.97	101.52
3	2.5	2.5	0.2	98.80	98.80	101.00	50.0	50.0	50.0	102.55	98.53	101.99	120.0	100.0	200.0	99.93	101.20	101.99
4	12.5	2.5	0.2	99.20	98.00	102.00	100.0	150.0	100.0	99.75	102.32	98.88	700.0	200.0	300.0	98.52	100.53	98.56
5^b^	24.0	1.44	0.3^c^	101.88	100.00	102.33d	50.0	33.0^c^	30.2^c^	100.41	99.95	99.63	1000.0	60.0	4.0	100.10	100.45	99.80
6	7.5	10.0	0.4	99.60	99.10	99.50	100.0	100.0	50.0	101.73	102.25	99.51	100.0	100.0	100.0	99.89	98.97	101.52
Mean ± SD				99.30 ± 1.484	99.23 ± 0.755	101.03 ± 1.470				100.87 ± 1.143	100.97 ± 1.470	100.33 ± 1.390				98.85 ± 0.831	100.02 ± 1.001	100.56 ± 1.328

^a^Average of three determinations

^b^Ratio present in dosage form

^c^Amount spiked was 0.204 µg/band of BRM for the TLC method and was 30.00 µg/mL of PSE and BRM for HPLC method

^d^After spiking and subtraction of the added standard

**Table 3:** Analysis of PAR, PSE and BRM in Comtrex^®^ maximum strength tablets and application of standard addition technique using the proposed chromatographic methods

Drug	TLC			HPLC–UV			UPLC-MS/MS			Reference methods [26, 29]
	Claimed amount taken	Added	Recovery %^a^	Claimed amount taken	Added	Recovery %^a^	Claimed amount taken	Added	Recovery %^a,b^	Recovery %^a^
PAR										
	10.0 (µg/band) [102.02^b^]	5.0	101.80	25.0 (µg/mL) [99.83^b^]	10.0	100.60	1000.0 (ng/mL)		101.33	102.16 100.22 102.80 103.00
		10.0	102.00		25.0	101.04			101.89	
		14.0	99.20		50.0	101.98			102.07	
	Mean ± SD		100.97 ± 1.56	Mean ± SD		101.20 ± 0.70	Mean ± SD		101.76 ± 0.386	Mean ± SD 102.55 ± 0.419
Student’s *t* test							2.52 (2.57)			
*F*-test							1.180 (9.550)			
PSE										
	3.0 (µg/band) [100.62^b^]	1.0	100.00	51.5 (µg/mL) [98.65^b^]	30.0	98.87	60.0 (ng/mL) [98.67^b^]	30.0	101.60	101.35 101.58 101.58 101.57
		3.0	98.00		51.5	98.62		60.0	101.85	
		10.0	97.60		100.0	97.41		200.0	101.79	
	Mean ± SD		98.50 ± 1.20	Mean ± SD		98.30 ± 0.78	Mean ± SD		101.75 ± 0.131	Mean ± SD 101.58 ± 0.006
Student’s *t*-test							2.46 (2.57)			
*F*-test							1.320 (9.550)			
BRM										
	0.2 (µg/band) [100.59^b^]	0.1	100.00	50.1 (µg/mL) [99.01^b^]	30.0	100.33	4.0 (ng/mL) [99.80^b^]	2.0	101.11	101.15 101.12 101.10 100.13
		0.2	100.00		50.1	99.84		4.0	101.16	
		1.0	99.00		100.0	98.67		100.0	101.17	
	Mean ± SD		99.67 ± 0.58	Mean ± SD		99.61 ± 0.85	Mean ± SD		101.15 ± 0.032	Mean ± SD 101.13 ± 0.021
Student’s *t*-test							1.09 (2.57)			
*F*-test							2.390 (9.550)			

^a^Average of three experiments

^b^Recovery of the claimed amount taken. Figures between parentheses represent the corresponding tabulated values of t and F at P = 0.05. Reported method for determination of PAR and PSE is an HPLC method using C_18_ column, a mobile phase composed of 25 mM phosphate buffer (pH = 5):methanol:acetonitrile (30:60:10, v/v/v) at flow rate 1 mL/min and detection at 240 nm. Reported method for determination of BRM is a TLC using methanol:ammonia (100:1.5 v/v) as mobile phase

#### Limit of detection (LOD) and limit of quantification (LOQ)

The LOD is the least concentration of the drug which could be reliably detected but not essentially quantified, using the described experimental procedures. The LOQ is the minimum concentration of the drug which could be measured with satisfactory accuracy and precision [30]. The LOD and LOQ were calculated using the following equations:

$$\begin{aligned} {\text{LOD}} & = 3. 3*{\text{S}}_{{{\text{y}}/{\text{x}}}} /{\text{slope of the calibration curve}} \\ {\text{LOQ}} & = 10*{\text{S}}_{{{\text{y}}/{\text{x}}}} /{\text{slope of the calibration curve}} \\ \end{aligned}$$

where S_y/x_ is the standard deviation of residuals. The results are abridged in Table 1.

#### Robustness

The robustness of the method was confirmed by the consistency of the peak area ratios of the drugs with the intended slight changes performed. For the TLC method, a small change was performed in the percentage of methanol ± 2%. For the HPLC–UV method, a deliberate change was conducted in pH 3.2 ± 0.2 and in the percentage of acetonitrile ± 2%. For the UPLC-MS/MS method a slight change was performed in the percentage of methanol ± 2%, capillary temperature ± 5 °C and collision energy ± 2 V. In all cases slight shifts was noticed in the retention times or retention factors however, the peak areas were usually remain unchanged proving the robustness of the proposed methods (Table 1).

### System suitability

The system suitability parameters including retention time (t_R_), retention factor (R_f_), tailing factor (T), selectivity factor (α), theoretical plate count (N), height equivalent to theoretical plate (HETP), and resolution (Rs) were all calculated in accordance to the United States Pharmacopeia (USP) guidelines [31]. The results demonstrated in Table 4 conform to the USP limits thus proving the good performance of the proposed methods.

**Table 4:** Calculation of the system suitability parameters required for testing of TLC and HPLC methods

Parameter	TLC			HPLC–UV			Reference value
	PSE	BRM	PAR	PSE	BRM	PAR	
Retention time (t_R_)				1.521	2.164	3.414	t_R_ > 1
Retention factor (R_f_)	0.24	0.32	0.81				
Column efficiency (N)				2846.3	2042.3	4788.5	N > 2000 Increases with efficiency of the separation
Height equivalent to theoretical plates (HETP)				5.27 × 10^−3^	7.34 × 10^−3^	3.13 × 10^−3^	The smaller the value, the higher the column efficiency
Selectivity factor (α)	1.51	2.48		1.44	1.56		α > 1
Tailing factor (T)	0.786	0.917	0.885	1.13	1.07	1.40	T < 2 T = 1 for symmetric peak
Resolution (R_s_)	2.10	8.70		4.13	6.02		R_s_ > 2

### Application of the proposed methods to the analysis of laboratory prepared mixture and Comtrex^®^ Maximum Strength tablets

The proposed methods were efficiently utilized for analyzing laboratory prepared mixtures consisting of different quantities of PAR, PSE and BRM. The average percentage recoveries were calculated based on the average of three determinations (Table 2). Comtrex^®^ Maximum Strength tablets were analyzed so as to prove the applicability of the proposed methods for the routine analysis of the drugs under investigation in their pharmaceutical formulation in quality control labs. The computed regression equations for each method were used for the calculation of the corresponding concentrations of the drugs. Also the standard addition technique was utilized in order to examine the effect of the frequently used excipients. The data obtained from the standard addition technique which was expressed as mean percentage recoveries and standard deviation indicated a satisfactory precision and accuracy for the proposed methods (Table 3).

Beside the analysis of the dosage form and due to the promising results acquired by the UPLC-MS/MS method a preliminary investigation was performed to examine the applicability of the proposed method in the analysis of plasma samples. Accordingly, plasma samples were spiked with various concentrations of the PAR, PSE and BRM using diphenhydramine as an internal standard. The drugs were extracted using liquid–liquid extraction by ethyl acetate, followed by evaporation of the organic layer and the residue was dissolved in methanol. The mean percentage recoveries of the drugs were calculated and were found to be 92.85% ± 1.23, 90.66% ± 1.34 and 91.55% ± 1.55 for PAR, PSE and BRM, respectively. These primary results assured the suitability of the UPLC-MS/MS method for determining PAR, PSE and BRM in spiked plasma samples. As a next step in the upcoming future; more optimization for the extraction procedure will be carried out, then quality control samples (QCs) will be prepared in order to perform a full validation for the method according to the recommendations of the FDA [32]. Additionally, plasma samples from healthy volunteers will be examined to evaluate the potential of the UPLC-MS/MS method for pharmacokinetic studies.

### Statistical analysis

Statistical comparison between the achieved results by both the proposed methods and reported ones [26, 29] was carried out. The chromatographic conditions were applied to the pure authentic powders of each drug separately. The results abridged in Table 5 demonstrates the absence of any significant difference between the proposed and reported methods considering the accuracy and precision as implied from the lower values of the calculated t and F tests than the tabulated values.

**Table 5:** Statistical comparison of the results obtained by the proposed methods and reference methods for the determination of PAR, PSE and BRM

	PAR			Ref. [29]	PSE			Ref. [29]	BRM			Ref. [26]
	TLC	HPLC	UPLC		TLC	HPLC	UPLC		TLC	HPLC	UPLC	
Mean	99.46	100.67	99.55	99.40	99.74	99.02	98.99	100.11	100.02	100.32	98.28	99.12
SD	1.569	1.430	1.270	0.778	1.090	1.171	1.11	0.427	0.735	1.521	0.910	0.699
N	6	6	6	4	6	6	6	4	6	6	6	4
Variance	2.462	2.045	1.613	0.605	1.188	1.371	1.232	0.182	0.540	2.310	0.828	0.489
Student’s t	0.080 (2.310)	1.611 (2.310)	0.374 (2.310)		0.640 (2.310)	1.762 (2.310)	1.870 (2.310)		1.920 (2.310)	1.464 (2.310)	1.586 (2.310)	
F	4.07 (9.01)	3.38 (9.01)	2.67 (9.01)		6.53 (9.01)	7.52 (9.01)	6.77 (9.01)		1.10 (9.01)	4.72 (9.01)	1.69 (9.01)	

Figures between parentheses represent the corresponding tabulated values of t and F at P = 0.05

Reported method for determination of PAR and PSE is an HPLC method using C_18_ column, a mobile phase composed of 25 mM phosphate buffer (pH = 5):methanol:acetonitrile (30:60:10, v/v/v) at flow rate 1 mL/min and detection at 240 nm

Reported method for determination of BRM is a TLC using methanol:ammonia (100:1.5 v/v) as mobile phase

## Conclusion

Three simple chromatographic methods were developed for the simultaneous determination of PAR, PSE and BRM in their pharmaceutical dosage form. The different techniques provide choices of apparatus according to their availability, also the methods are characterized by the utility of economic solvents which are readily available in any quality control laboratory. The proposed methods are considered rapid with a run time ranging between 1 min for UPLC-MS/MS, and 4 min for HPLC thus lowering solvent consumption which is a privilege from the economic and eco-friendly points of view. The obtained results are of good sensitivity specifically the UPLC-MS/MS method which was capable of determining the drugs in the nano-gram level. This study can be used as a preliminary stage for the development of an assay of the three drugs in human plasma. Validation was performed according to the ICH guidelines where the results are linear, accurate, precise, specific and robust. Based on the previous advantages and results all the developed methods can be appropriately used by quality control laboratories.

## Additional file

**Additional file 1.** Optimization conditions of the developed HPLC method.

## Data Availability

Some of the chromatograms for optimization conditions are available in the additional file. Otherwise, all data is included in the manuscript.

## Abbreviations

BRM: brompheniramine maleate
GC: gas chromatography
HPLC–UV: high performance liquid chromatography-ultra violet detection
MECC: micellar electrokinetic capillary chromatography
PAR: paracetamol
PSE: pseudoephedrine hydrochloride
TLC: thin layer chromatography
UPLC-MS/MS: ultra performance liquid chromatography-tandem mass spectrometry
