# Supplementary material for: Analytical methods for the determination of paracetamol, pseudoephedrine and brompheniramine in Comtrex tablets
Source: BMC Chem. 2019 Jul 2;13(1):78. doi: 10.1186/s13065-019-0595-6 (PMC6661757; doi:10.1186/s13065-019-0595-6)
Supplement: Supplementary file 1 — Additional file 1. Optimization conditions of the developed HPLC method. [file 13065_2019_595_MOESM1_ESM.docx]

**Table (S1): Optimization of the proposed mobile phase in TLC method**

| **Parameter** | **TLC method** | | | | | | |
| --- | --- | --- | --- | --- | --- | --- | --- |
|  | PSE | BRM | | PAR | PSE | BRM | PAR |
| **Mobile Phase**  (Methanol: Water: Ammonia, v/v/v) | **Resolution (R_s_)**  Reference value: Rs > 1.5 | | | | **Tailing**  Reference value = T < 2  T = 1 for symmetric peak | | |
| **8.5: 1.5 : 0.1** | 2.20 | | 8.65 | | 2.10 | 1.10 | 0.950 |
| **9: 1: 0.1*** | 2.10 | | 8.70 | | 0.786 | 0.917 | 0.885 |
| **9.5: 0.5: 0.1** | 2.05 | | 8.85 | | 0.790 | 0.95 | 2.10 |
| **10: 0: 1** | 2.30 | | 9.24 | | 0.820 | 0.852 | 2.50 |

*Optimum mobile phase ratio

**Table (S2): Optimization of detection wavelength in TLC method**

| **Parameter** | **TLC method** | | | | |
| --- | --- | --- | --- | --- | --- |
|  | PSE | BRM | | PAR | |
| **Wavelength (nm)**  Mobile phase =  Methanol: Water: Ammonia, v/v/v  9: 1: 0.1 | **Peak Area^a^** | | | | |
| **210** | 1129.8 | | 1056.8 | | 1159.4 |
| **230** | 688.0 | | 859.2 | | 1058.6 |
| **254*** | 1819.4 | | 1259.5 | | 1298.7 |
| **265** | 426.5 | | 352.8 | | 472.7 |

* Optimum detection wavelength

^a^ Lab prepared mixture composed of 1.00 μg/band PSE, 1.00 μg/band BRM and 1.00 μg/band PAR.

**Table (S3): Optimization of the proposed mobile phase in HPLC method**

| **Parameter** | **HPLC-UV method** | | | | | | |
| --- | --- | --- | --- | --- | --- | --- | --- |
|  | PSE | BRM | | PAR | PSE | BRM | PAR |
| **Mobile Phase**  (Water: Acetonitrile, v/v)  pH=3.2  Flow rate = 0.7 mL/min | **Resolution (R_s_)**  Reference value: Rs > 1.5 | | | | **Tailing**  Reference value = T < 2  T = 1 for symmetric peak | | |
| **65:35** | 1.3 | | 1.13 | | 1.75 | 2.04 | 2.35 |
| **70:30** | 1.45 | | 1.52 | | 1.82 | 2.05 | 2.20 |
| **75:25*** | 4.13 | | 6.02 | | 1.13 | 1.07 | 1.40 |
| **72:28** | 1.58 | | 7.42 | | 1.50 | 1.65 | 1.55 |
| **74:26** | 4.12 | | 6.00 | | 1.18 | 1.05 | 1.38 |

*Optimum mobile phase ratio

**Table (S4): Optimization of pH in HPLC method**

| **Parameter** | **HPLC-UV method** | | | | |
| --- | --- | --- | --- | --- | --- |
|  | PSE | BRM | | PAR | |
| **pH**  Mobile phase =  Water: Acetonitrile, v/v;  75:25  Flow rate = 0.7 mL/min | **Tailing**  Reference value = T < 2  T = 1 for symmetric peak | | | | |
| **3*** | 1.13 | | 1.07 | | 1.40 |
| **5** | 1.52 | | 2.35 | | 2.42 |
| **8** | 1.59 | | 2.84 | | 2.57 |

*Optimum mobile pH.

**Table (S5): Optimization of flow rate in HPLC method**

| **Parameter** | **HPLC-UV method** | | | | | | | |
| --- | --- | --- | --- | --- | --- | --- | --- | --- |
|  | PSE | BRM | | PAR | | PSE | BRM | PAR |
| **Flow rate (mL/min)**  Mobile phase =  Water: Acetonitrile, v/v; 75:25  pH = 3.2 | **Tailing**  Reference value = T < 2  T = 1 for symmetric peak | | | | | **Retention Time**  **(min)** | | |
| **1.0** | 1.22 | | 1.15 | | 1.48 | 1.102 | 1.850 | 2.985 |
| **0.7*** | 1.13 | | 1.07 | | 1.40 | 1.521 | 2.164 | 3.414 |
| **0.5** | 1.60 | | 2.05 | | 1.88 | 1.951 | 2.854 | 4.021 |

*Optimum flow rate.

**Table (S6): Optimization of detection wavelength in HPLC method**

| **Parameter** | **HPLC-UV method** | | | | |
| --- | --- | --- | --- | --- | --- |
|  | PSE | BRM | | PAR | |
| **Wavelength (nm)**  Mobile phase =  Water: Acetonitrile, v/v;  75:25)  pH = 3.2  Flow rate = 0.7 mL/min | **Column efficiency (N)**  N > 2000 | | | | |
| **210*** | 2846.3 | | 2042.3 | | 4788.5 |
| **230** | 1058.4 | | 1999.8 | | 2568.2 |
| **254** | 2541.2 | | 2001.5 | | 2589.2 |
| **265** | 985.2 | | 598.5 | | 552.9 |

* Optimum detection wavelength
